# Contrasting Pediatric and Adult Methicillin-resistant *Staphylococcus aureus* Isolates

**DOI:** 10.3201/eid1204.050960

**Published:** 2006-04

**Authors:** Michael Z. David, Susan E. Crawford, Susan Boyle-Vavra, Mark A. Hostetler, Daniel C. Kim, Robert S. Daum

**Affiliations:** * University of Chicago, Chicago, Illinois, USA

**Keywords:** Methicillin resistance, Staphylococcus aureus, clindamycin, drug resistance, research

## Abstract

We performed a prospective study of all inpatient and outpatient methicillin-resistant *Staphylococcus aureus* (MRSA) isolates identified at the University of Chicago Hospitals from November 2003 through November 2004. Differences in resistance to non–β-lactam antimicrobial drugs were determined after stratification of the 578 MRSA isolates into 4 groups by patient age (pediatric vs. adult) and onset location (community vs. hospital). Non–β-lactam resistance was significantly greater among the 288 adult than the 177 pediatric community-associated isolates for erythromycin (93.2 vs. 87.0%, p = 0.03), clindamycin (51.8 vs. 7.3%, p<0.001), ciprofloxacin (62.1 vs. 10.7%, p<0.001), gentamicin (11.1 vs. 1.1%, p<0.001), and tetracycline (19.9 vs. 6.4%, p<0.001). In contrast, hospital-associated MRSA isolates from children and adults had similar rates of non–β-lactam antimicrobial drug resistance. In our region, clindamycin is an appropriate empiric therapy of community-associated MRSA infection in children but should be used with caution in adults.

## Abstract 2

Children may share a reservoir of MRSA strains that have an antimicrobial drug resistance profile distinct from that of adults.

Colonization by and infection with methicillin-resistant *Staphylococcus aureus* (MRSA) in children and adults who have little or no contact with the healthcare system, phenomena almost unknown before the mid-1990s, have been reported with increasing frequency (1–34). In some cities and rural areas, rates of these community-associated MRSA (CA-MRSA) infections, particularly in skin and soft tissue, are increasing rapidly (*1**–**4*). Many CA-MRSA infections have been attributed to isolates that are distinct from hospital-associated MRSA (HA-MRSA) strains.

The definition of CA-MRSA has not been standardized. No single definition would likely suffice for all purposes. Attempts to define CA-MRSA have focused upon a variety of criteria: temporal (i.e., the MRSA isolate from a specimen is submitted ≈72 hours after hospital admission), host-risk-factor profile (i.e., the patient with the MRSA isolate lacked certain exposure parameters relevant to healthcare facilities, variously defined), antimicrobial drug susceptibility of the MRSA isolates (e.g., susceptibility to clindamycin), and certain molecular aspects of the isolates (e.g., SCC*mec* type or pulsed-field gel electrophoresis type). Which definition is most important in the choice of empiric antimicrobial drug treatment is not clear. However, a clinician confronted with a putative *S. aureus* infection needs guidance.

The epidemiology of the emerging CA-MRSA strains, including their origins, reservoirs, modes of dissemination, and effective approaches to their control, is not completely understood. We previously noted that CA-MRSA isolates often lacked resistance to multiple non–β-lactam antimicrobial drugs when compared with HA-MRSA strains (*5**,**6*). This phenomenon has been documented in adults (*4**,**7*) and children (*8**–**10*). More recently, we anecdotally observed that children had MRSA infections caused by strains susceptible to non–β-lactam antimicrobial drugs more often than adults. Few data exist that compare antimicrobial drug resistance profiles of adult and pediatric MRSA infection isolates stratified by site of onset (hospital vs. community). Accordingly, using the temporal definition of CA-MRSA, we examined the hypothesis that these 2 groups may be infected by MRSA strains with different rates of resistance to non–β-lactam antimicrobial drugs when the isolates had onset in the community. Therefore, we compared several criteria for the temporal definition of CA-MRSA to assess the impact of changing these criteria on our findings. This article summarizes our study.

## Materials and Methods

The University of Chicago Hospitals (UCH) is a tertiary-care medical center on the south side of Chicago with 577 inpatient beds and 29,500 annual admissions. It includes an outpatient care facility with 379,000 annual visits. UCH serves an inner city population and draws tertiary referrals from the surrounding region.

The UCH Clinical Microbiology Laboratories prospectively identified all MRSA isolates collected at the medical center from November 7, 2003, to November 7, 2004, from inpatients and outpatients. *S. aureus* isolates were identified by gram stain, growth on BBL mannitol salt fermentation agar (Beckton, Dickinson and Company, Sparks, MD, USA), positive catalase test results, and a positive Staphaurex Plus test (Remel Europe Ltd., Dartford, UK) result.

The Clinical Microbiology Laboratories determined the susceptibility profile of each isolate by using the Vitek 2 system (bioMérieux Vitek, Inc., Durham, NC, USA) for methicillin, erythromycin, clindamycin, ciprofloxacin, rifampin, gentamicin, tetracycline, and vancomycin. From November 7, 2003, to July 2004, any isolate with an MIC of vancomycin >2 μg/mL reported by the Vitek system was retested for vancomycin susceptibility by the E test Strip (AB Biodisk, Solna, Sweden). After July 2004, the E test was performed for these isolates only if growth occurred on a vancomycin agar screen plate. For isolates that tested resistant to erythromycin but susceptible to clindamycin, a D-test to detect inducible resistance to clindamycin was performed, as was a Kirby-Bauer disk-diffusion test for susceptibility to trimethoprim-sulfamethoxazole (TMP-SMX), both according to Clinical and Laboratory Standards Institute (CLSI) guidelines (*35*).

For each MRSA specimen, patient information was collected from the laboratory information system and the electronic medical record, including the date of specimen procurement, age and sex of the patient, location of specimen procurement (inpatient, outpatient, emergency department), and antimicrobial drug susceptibility profile. For inpatient isolates, the number of days from admission to culture procurement was determined by subtracting the admission date from the procurement date.

We defined CA-MRSA to include all isolates cultured from outpatients and isolates from hospitalized patients obtained within 72 hours of admission. We also examined the impact of changing the 72-hour criterion in the definition to 24 and 48 hours. With each change in definition, the effect on the percentage of MRSA infections considered to be community associated was assessed.

The bacteriologic and patient information was compiled in an electronic database designed for the study by using Access software (Microsoft, Redmond, WA, USA). Only the first isolate from each patient collected during the surveillance was included in the database. The study was approved by the institutional review board of the Biological Sciences Division of the University of Chicago. Data were analyzed with Stata, version 8.0 (Stata Corp, College Station, TX, USA). Comparisons between groups were performed by the χ^2^ test or the Fisher exact test. All hypotheses were 2-tailed and were considered significant if p<0.05.

## Results

The UCH Clinical Microbiology Laboratories identified 1,149 MRSA-positive cultures from 578 patients. Of these, 201 (34.7%) were from children <18 years of age and 377 (65.3%) were from adults >19 years of age. Of the adult MRSA isolates, 27.9% were cultured from outpatients; of the pediatric MRSA isolates, 57.2% were cultured from outpatients. The median age of pediatric patients was 3 years (16 days to 18 years), with a median of 6 years among outpatients and 2 years among inpatients. For adults, the median age was 56 years (range 19–100), with a median of 51 years among outpatients and 60 years among inpatients.

Using the 72-hour definition, 64.3% of adult and 72.1% of pediatric inpatient isolates were CA-MRSA (p = 0.2) (Table 1). Restricting the definition of CA-MRSA to the 48-hour definition, 58.5% of adult and 69.8% of pediatric inpatient isolates were CA-MRSA (p = 0.06). Fifty-four percent and 65.1%, respectively, of adult and pediatric inpatient isolates met the criteria for the 24-hour definition of CA-MRSA (p = 0.07). Thus, by using the 24-, 48-, or 72-hour criteria for CA-MRSA, most of the pediatric and adult inpatient isolates would be considered community-associated.

**Table 1:** Inpatient adult and pediatric methicillin-resistant *Staphylococcus aureus* isolates by procurement time after admission*

Time after admission, h	Adult inpatient isolates (%), n = 272	Pediatric inpatient isolates (%), n = 86	p value†
<24	147 (54.0)	56 (65.1)	0.07
<48	159 (58.5)	60 (69.8)	0.06
<72	175 (64.3)	62 (72.1)	0.18

*Values are number (%). †p value comparing adult and pediatric isolates for each category of time after admission, χ^2^ test.

Combining the isolates cultured in the emergency department with those cultured in clinics, 105 outpatient isolates were obtained from adults and 115 from children. Adult isolates were substantially more likely than pediatric isolates to be resistant to ciprofloxacin, clindamycin, gentamicin, and tetracycline. No difference was seen in resistance rates for erythromycin or rifampin (Table 2).

**Table 2:** Percentage of methicillin-resistant *Staphylococcus aureus* isolates resistant to non–β-lactam antimicrobial drugs among pediatric and adult patients, stratified by hospital- and community-associated designation

Antimicrobial drug	Adult outpatient isolates (n = 105)	Pediatric outpatient isolates (n = 115)	p value*
Ciprofloxacin	55.2	9.6	<0.001
Clindamycin			
Resistant†	37.1	6.1	<0.001
D-test positive‡	20.4	14.8	0.39
Erythromycin	91.4	88.7	0.16
Gentamicin	8.6	1.7	0.03
Rifampin	1.9	0	0.23
Tetracycline§	13.3	3.6	0.01
TMP-SMX¶	0	0	NA
Vancomycin	0	0	NA

*p value compares resistance to indicated antimicrobial drugs or positive test result among adult vs. pediatric isolates by χ^2^ test or Fisher exact test. NA, not applicable. †Figures in this row represent Vitek testing results for clindamycin. ‡54 (97.1%) and 88 (93.9%) of the adult and pediatric isolates, respectively, that were erythromycin resistant and clindamycin susceptible by Vitek were evaluated by D-testing. §Tetracycline susceptibility was not tested for 5 pediatric outpatient isolates and 2 adult outpatient isolates. ¶TMP-SMX, trimethoprim-sulfamethoxazole. Only 42 adult and 27 pediatric isolates that were erythromycin resistant and clindamycin susceptible were tested.

A small percentage of the temporally defined CA-MRSA isolates (i.e., those either from outpatients or cultured <72 hours after admission from inpatients) was resistant only to β-lactam antimicrobial drugs; this resistance pattern was more common among pediatric (9.6%) than adult (3.6%) isolates (p = 0.001) (Table 3). Among the temporally defined CA-MRSA isolates, those from adults were more likely than those from children to be resistant to most of the non–β-lactam antimicrobial drugs. The rate of resistance to erythromycin was high among both the pediatric and adult isolates, although significantly higher among the adult isolates. The rate of resistance to clindamycin, ciprofloxacin, gentamicin, and tetracycline was much higher for adult than for pediatric CA-MRSA isolates (Table 3). There was no significant difference for rifampin, TMP-SMX, or vancomycin.

**Table 3:** Percentage of methicillin-resistant *Staphylococcus aureus* (MRSA) resistant to non–β-lactam antimicrobial drugs among pediatric and adult patients, stratified by hospital- and by community-associated designation

In addition to methicillin,* % resistant to	Adult community-associated, % (n = 280)	Pediatric community-associated, % (n = 177)	p value†	Adult hospital-associated, % (n = 97)	Pediatric hospital-associated, % (n = 24)	p value‡
No other antimicrobial drugs§	3.6	9.6	0.001	3.1	4.2	0.99
Ciprofloxacin	62.1	10.7	<0.001	87.6	62.5	0.004
Clindamycin						
Resistant¶	51.8	7.3	<0.001	74.2	75.0	0.94
D-test positive#	20.5	15.2	0.29	50	0	0.48
Erythromycin	93.2	87.0	0.03	93.8	95.8	0.99
Gentamicin	11.1	1.1	<0.001	14.4	37.5	0.01
Rifampin	1.8	0	0.16	1.0	0	0.99
Tetracycline**	19.9	6.4	<0.001	13.5	8.3	0.73
TMP-SMX††	0	0	NA	0	0	NA
Vancomycin	0	0	NA	0	0	NA
>3 non–β-lactam antimicrobial drugs	52.3	6.4	<0.001	76.0	66.7	0.35

*Methicillin resistance inferred from oxacillin resistance test. †p value compares community-associated (CA) adult and pediatric isolates for resistance to each antimicrobial drug or test, χ^2^ or Fisher exact test. NA, not applicable. ‡p value compares hospital-associated (HA) MRSA adult and pediatric isolates for resistance to each antimicrobial drug or test, χ^2^ or Fisher exact test. §Includes erythromycin, clindamycin, ciprofloxacin, gentamicin, rifampin, and tetracycline and does not include D-test–positive results. ¶Data in this row represent single-agent Vitek testing results for clindamycin. #112 (97%) of the adult CA-MRSA isolates, 125 (89%) of the pediatric CA-MRSA isolates, 18 (95%) of the adult HA-MRSA isolates, and 2 (67%) of the pediatric HA-MRSA isolates that were erythromycin resistant and clindamycin susceptible by Vitek were evaluated by D-testing. **Nine isolates not tested for susceptibility to tetracycline were omitted. ††TMP-SMX, trimethoprim-sulfamethoxazole. Only 100 adult CA-MRSA, 121 pediatric CA-MRSA, 15 adult HA-MRSA, and 2 pediatric HA-MRSA isolates that were clindamycin susceptible and erythromycin resistant were tested for TMP-SMX susceptibility.

The D-test for inducible clindamycin resistance was performed on 97% (112/116) of adult and 89% (125/142) of pediatric CA-MRSA isolates that were resistant to erythromycin and susceptible to clindamycin. Among those tested, adult (20.5%, 23/112) isolates were more likely than pediatric (15.2%, 19/125) isolates to have a positive D-test result (Table 3).

In contrast to the differences we found among CA-MRSA isolates from adults and children, however, the hospital-associated MRSA isolates (>72 hours after admission) had similar rates of antimicrobial drug resistance (Table 3). For example, clindamycin resistance occurred in 74.2% of adult and 75.0% of pediatric HA-MRSA isolates (p = 0.9). Only the rates of ciprofloxacin (p = 0.004) and gentamicin (p = 0.01) resistance were substantially different when the HA-MRSA isolates were compared (Table 3).

We defined a multidrug-resistant (MDR) MRSA isolate as being resistant to >3 of the non–β-lactam antimicrobial drugs tested. When the temporal definition (<72 hours after admission) was used, adult CA-MRSA isolates were more likely than pediatric CA-MRSA isolates (52.3% vs. 6.4%, p<0.0001) to be MDR (Table 3). HA-MRSA isolates from both children and adults were more often MDR, but the rates did not differ substantially (76% vs. 66.7%, p = 0.4) (Table 3).

## Discussion

When community association or onset was defined by using the temporal criterion of procuring isolates <72 hours after admission, most adult and most pediatric MRSA isolates in our study would be considered to have a community origin. A narrower procurement definition, e.g., <24 hours or <48 hours, would have led to the same conclusion.

MRSA isolates from children's specimens compared with isolates from adults' specimens obtained <72 hours after admission were more likely to be resistant to only β-lactams and more likely to be susceptible to clindamycin, ciprofloxacin, and gentamicin. These differences suggest that there may be distinct CA-MRSA isolates colonizing and infecting children and adults in the population served by our medical center or, less likely, that there may be a reservoir of CA-MRSA that affects children and adults differently.

Children may have unique risk factors for MRSA colonization in the community related to their environment, such as daycare centers (*11**,**12*), schools (*13*), or recreational facilities, and may have different behavioral habits than most adults. Alternatively, children may be different from adults as hosts, and they may encounter novel MRSA strains either by different colonization of the skin or by some undefined difference in host defense. Antimicrobial drugs used to treat infections among children may differ from those used among adults; perhaps distinct MRSA strains colonizing children result from differential antimicrobial drug selection pressures in the community.

Our findings have obvious implications for empiric therapy of CA-MRSA infections and MRSA control measures. In the population served by UCH, for children with suspected community-onset MRSA infections, clindamycin is an appropriate first-line empiric antimicrobial drug, at least for those who are not critically ill (*8**,**10*). In adults, by contrast, clindamycin would be much less suitable in this role, as most temporally defined CA-MRSA isolates are clindamycin-resistant. Therefore, among adults at our institution, clindamycin can be considered only after susceptibility testing results have been obtained from a culture specimen.

CA-MRSA isolates from children have a low rate of clindamycin resistance. Studies of children with CA-MRSA infections or colonization in many cities, including Houston, Corpus Christi, Memphis, Nashville, Louisville, Providence, and Chicago, have demonstrated high rates of clindamycin susceptibility among these isolates, ranging from 67%–100% (*1**,**8**,**10**,**14**–**16*). The 2 studies with the lowest susceptibility rates were conducted in Providence from 1997 to 2001 with a 74% rate (*8*), and Louisville from 1999 to 2001 with a 67% rate (*16*). These lower rates may have been due to geographic variation or were a window on an earlier phase in the CA-MRSA epidemic.

A possible confounder in calculating clindamycin resistance rates is the 2004 CLSI guideline stating that each isolate that initially tests susceptible to clindamycin and resistant to erythromycin should be tested for inducible clindamycin resistance by the D-test; if the test result is positive, that isolate should be considered resistant (*35*). Published data from before 2004 may not have included D-test results in reporting clindamycin resistance rates among MRSA isolates. However, the impact of the new CLSI guideline does not change the conclusions derived from our data, although the rate of clindamycin resistance among all pediatric MRSA isolates in our sample would increase from 15.4% to 24.9% when the new guideline was considered.

We have continued to use clindamycin for initial empiric therapy of mild or moderately ill pediatric patients likely to have a CA-MRSA infection. We take this approach because better alternative therapy is not available. The results of TMP-SMX therapy for CA-MRSA have been reported for relatively few patients (*36*); therapy was frequently unsuccessful despite in vitro susceptibility of the infecting MRSA strains. Tetracyclines are not suitable for young children, and linezolid is prohibitively expensive. Our policy is to abandon clindamycin when susceptibility testing or D-test results suggest the possibility of treatment failure, usually 2–4 days into the treatment course or, as mentioned, for severe illness when CA-MRSA is suspected. To date, this approach has been suitable.

The pattern of clindamycin resistance among adult CA-MRSA isolates has been more complex. At UCH, more than half of the CA-MRSA isolates from adults were resistant to clindamycin. Similarly, at Northwestern Memorial Hospital, also in Chicago, from 1998 to 1999, few MRSA isolates were clindamycin susceptible among those that were collected <72 hours after admission from adults with no known hospitalizations in the previous 2 years (*37*).

In contrast, more recent studies of CA-MRSA infections and MRSA colonization isolates have demonstrated low rates of clindamycin and other non–β-lactam resistance among MRSA isolates from young and urban poor adults. For example, 9 of 67 patients with MRSA infections in a military-beneficiary population in Texas from 1999 to 2001 had no risk factors for HA-MRSA. These patients all had onset of infection in the community and had an isolate that was susceptible to clindamycin. Moreover, they were younger than the patients reported to have HA-MRSA infections (*4*). In San Francisco in 1999–2000, among asymptomatic, homeless and marginally housed adults in the community, 23 MRSA isolates were identified from nasal swabs of which only 2 (8.7%) were resistant to clindamycin (*17*). In a sample of 45 colonizing and infecting MRSA isolates collected from healthy young adults (18–44 years of age) training at a military facility in Texas, 22% were resistant to clindamycin (*7*).

Why these disparities exist in the rates of clindamycin susceptibility among CA-MRSA isolates obtained from adults is unclear. Possible explanations include regional variation or characteristics of the patient populations sampled. The 2 studies with high clindamycin resistance rates were both conducted in Chicago at tertiary medical centers. Community-based surveys among adults, in contrast, have demonstrated a high rate of clindamycin susceptibility, perhaps reflecting an exposure to a community MRSA isolate pool similar to the pool affecting pediatric populations in many urban centers.

Unlike clindamycin, erythromycin resistance has been common among both pediatric and adult CA-MRSA isolates. In hospitalized children with MRSA infections at UCH during 1988–1990 and 1993–1995, 74.3% carried isolates resistant to erythromycin (*5*). Our current rate, 88.1% in pediatric isolates, is slightly higher. Erythromycin resistance has also been common among CA-MRSA isolates colonizing and infecting children at many urban centers from 1990 to 2002, with rates from 52% to 100% (*8**,**10**,**14**,**16*). In adults, recent studies have shown similarly high rates of erythromycin resistance, 60.9%–80%, among CA-MRSA strains in selected populations, including soldiers (*7*), inmates in jail (*38*), and homeless and impoverished urban adults (*17*).

Analysis of antimicrobial resistance patterns among MRSA isolates obtained >72 hours after admission produced a sharp contrast with the CA isolates. Children and adults in this HA group were infected by MRSA strains with similar rates of resistance to non–β-lactam antimicrobial drugs. This finding suggests that adults and children face a common source of antimicrobial drug selection pressure and a common reservoir of MRSA isolates in our hospital, but not in the community.

Before this study, few data have been available to compare the resistance patterns of MRSA isolates collected in 1 medical center or region stratified by both age group (children vs. adults) and venue of onset (community vs. hospital). One previous analysis conducted from 1988 to 1997 at another medical center in Chicago compared adult and pediatric MRSA isolates and also found that MRSA strains susceptible to clindamycin were more common among pediatric than adult isolates; the isolates were not stratified by hospital or community origin (*18*). In contrast, among CA-MRSA isolates collected during 2000 from 12 Minnesota clinical microbiology laboratories serving both inpatients and outpatients, no differences in the rate of clindamycin susceptibility were found in isolates from children and adults (*19*). These investigators used a risk–factor-based definition for CA-MRSA, while we used a temporal definition. Other possible explanations for the discrepancy include a secular change in the 4 years separating the studies or demographic differences in the study populations.

Our study has certain limitations. It was conducted in 1 center and in 1 city, and it is unknown whether our data are representative of the CA-MRSA epidemic in other areas. Our study also assumes that adults and children seeking care in all areas of our medical center had an approximately equal chance of having a culture performed. We did not evaluate whether an assessment of risk factors for HA-MRSA would have determined the likelihood of resistance to non–β-lactam antimicrobial drugs.

With the recognition of CA-MRSA as a distinct epidemiologic phenomenon and the accompanying upsurge in both the *S. aureus* disease burden and methicillin resistance rates at many centers, considerable controversy has surrounded the definition most appropriate for CA-MRSA. Whether the best definition should be based on temporal, molecular, antimicrobial drug susceptibility, or host risk-factor criteria will not be resolved by this study and will continue to vary according to the issue being addressed. Despite these uncertainties, our data, using the temporal definition, have practical value in the initial antimicrobial drug management of patients with suspected CA-MRSA infections.

Understanding the complex epidemiology of CA-MRSA infection is critical to the development of control policies and treatment guidelines in areas with a high colonization and disease prevalence. Our data demonstrate the complexity of the contrasting definitions of CA-MRSA. While neither a time criterion alone nor a time criterion in addition to an antimicrobial drug susceptibility profile is adequate to define CA-MRSA, our data provide valuable guidance for clinical practice.
